# Environmental enrichment has minimal effects on behavior in the Shank3 complete knockout model of autism spectrum disorder

**DOI:** 10.1002/brb3.1107

**Published:** 2018-10-13

**Authors:** Samuel W. Hulbert, Alexandra L. Bey, Yong‐hui Jiang

**Affiliations:** ^1^ Department of Neurobiology Duke University School of Medicine Durham North Carolina; ^2^ Department of Pediatrics Duke University School of Medicine Durham North Carolina; ^3^ Duke Institute of Brain Science Duke University School of Medicine Durham North Carolina

**Keywords:** autism, autism spectrum disorder, environmental enrichment, mouse behavior, mouse models, Shank3

## Abstract

**Introduction:**

Several studies have supported the use of enriched environments to prevent the manifestation of ASD‐like phenotypes in laboratory rodents. While the translational value of such experiments is unknown, the findings have been relatively consistent across many different models.

**Methods:**

In the current study, we tested the effects of early environmental enrichment on a mouse model of ASD with high construct validity, the Shank3 ∆e4–22 mice our laboratory previously generated and characterized.

**Results:**

Contrary to previous reports, we found no benefits of enriched rearing, including no change in repetitive self‐grooming or hole‐board exploration. Instead, we found that early environmental enrichment increased anxiety‐like behavior in all mice regardless of genotype and decreased motor performance specifically in wild‐type mice.

**Conclusions:**

Although using a different enrichment protocol may have rescued the phenotypes in our mouse model, these results suggest that a “one‐size fits all” approach may not be the best when it comes to behavioral intervention for ASD and underscores the need for effective pharmaceutical development in certain genetic syndromes with severe symptom presentation.

## 1 INTRODUCTION

Currently, no pharmaceutical compound is approved to alleviate the core symptoms of autism spectrum disorder (ASD): restricted, repetitive behaviors, and impaired social communication. Early behavioral intervention has led to long‐lasting improvements in human patients with ASD (e.g., Rogers et al., 2012; Estes et al., 2015). Attempts to use sensorimotor enrichment on human patients based on findings in rodents have also reported some initial success (Woo & Leon, 2013; Woo, Donnelly, Steinberg‐Epstein, & Leon, 2015). However, these studies often exclude participants with known genetic conditions, warranting further investigation into interventions that can improve outcomes for individuals with genetic syndromes, who often have the most severe symptom presentation.

Numerous animal models have been created to tease apart the complex pathophysiology of ASD (Bey & Jiang, 2014; Hulbert & Jiang, 2016, 2017). One interesting finding from across various models is that housing rodents in an enriched environment, including more space and objects in which to interact and in some cases more rodents, prevents the expression of ASD‐like behavioral phenotypes (Favre et al., 2015; Garbugino, Centofante, & D'Amato, 2016; Kerr, Silva, Walz, & Young, 2010; Kondo et al., 2008; Lacaria, Spencer, Gu, Paylor, & Lupski, 2012; Lonetti et al., 2010; Nag et al., 2009; Oddi et al., 2015; Restivo et al., 2005; Reynolds, Urruela, & Devine, 2013; Schneider, Turczak, & Przewłocki, 2006; Yamaguchi et al., 2017). Although the type of enrichment and the developmental stages during exposure varied considerably, all these previous studies reported improvements on at least one behavioral outcome and seldom reported any adverse effects of enrichment. Moreover, some of the models utilized previously lack construct validity in terms of a known, highly penetrant cause of ASD, such as a genetic mutation that has been consistently linked to the disorder in humans.

Among the most prevalent genetic contributors to ASD are mutations in and deletions of *SHANK3* (Boccuto et al., 2013; Durand et al., 2007; Moessner et al., 2007). At least fourteen different lines of *Shank3* germline mutant mice have been reported (Bozdagi et al., 2010; Duffney et al., 2015; Jaramillo et al., 2017, 2016 ; Kouser et al., 2013; Lee et al., 2015; Mei et al., 2016; Peça et al., 2011; Schmeisser et al., 2012; Speed et al., 2015; Wang et al., 2016, 2011 ; Zhou et al., 2016). The expression of ASD‐like behaviors in each of these lines of mice confirms an important role for Shank3 in shaping behavior, but the majority of ASD patients with *SHANK3* mutations are missing the entire gene, so the ∆e4–22 mice, which lack all protein isoforms, have the greatest construct validity for the human condition (Wang et al., 2016). One group demonstrated that behaviors observed in *Shank3* exons 4–9 deletion (Δe4–9) mice are highly penetrant across different genetic backgrounds (Drapeau, Dorr, Elder, & Buxbaum, 2014), which contrasts findings in mice lacking the gene underlying fragile X syndrome, *Fmr1* (Baker et al., 2010; Dobkin et al., 2000; Errijgers, Fransen, D'Hooge, De Deyn, & Kooy, 2008; Pietropaolo, Guilleminot, Martin, D'Amato, & Crusio, 2011; Spencer et al., 2011). However, to date, no study has explored the role of the environment on behavioral phenotypes in *Shank3* mutant mice.

In the present study, we tested the effect of early environmental enrichment on some of the most robust behavioral phenotypes that we previously reported in our complete *Shank3* knockout model (∆e4–22) of ASD. Although ASD involves impaired social communication, we did not observe a strong social phenotype in our previous characterization of these mice using available behavioral assays (Wang et al., 2016) and therefore focused on repetitive behaviors and comorbidities, such as anxiety‐like behavior and motor impairments. We found that, contrary to previous findings using other mouse models of ASDs, early environmental enrichment did not prevent the manifestation of behaviors that resemble repetitive behaviors in *Shank3* ∆e4–22 mice: self‐grooming and restricted head poking on the hole‐board task. We also found that enrichment decreased motor performance on the rota‐rod task specifically in wild‐type mice and increased anxiety‐like behavior in all mice, regardless of genotype.

## 2 MATERIALS AND METHODS

### 2.1 Animals

*Shank3* ∆e4–22 mice were previously generated and characterized by our laboratory and were maintained on a C57BL/6J background after backcrossing for at least eight generations (Wang et al., 2016). All experiments were conducted with protocols approved by the Institutional Animal Care and Use Committee at Duke University.

### 2.2 Rearing conditions

Similar to a previous study utilizing a mouse model of Rett syndrome, mice were placed in enriched environment early in development (starting at postnatal day 10, P10) in an attempt to maximize effectiveness of the enrichment paradigm (Lonetti et al., 2010). In the enrichment condition, starting at P10, mice were housed with two lactating dams and two litters per cage, whereas in the standard condition, mice were housed with one lactating dam and one litter per cage. The enriched cages were modeled after those depicted in Ref.Laviola, Hannan, Macrì, Solinas, and Jaber (2008). They were larger than the standard mouse cage (approximately 30 × 15 × 15 cm) with approximate dimensions of 75 × 45 × 25 cm. Moreover, mice in the enriched environments had running wheel access as well as various shelters and objects in which to interact. The positions of the objects, shelters, and running wheels were rotated daily to maintain novelty, and the items were completely changed out weekly. Once the mice reached weaning age (P21), they were housed with same‐sex cage mates of seven per enriched cage or five per standard cage. Immediately prior to behavioral testing (starting once all mice in each cohort reached P60), all mice were transferred to standard cages so that the experimenter was blind to both genotype and rearing conditions.

### 2.3 Behavioral testing

Three cohorts of approximately 45 *Shank3* ∆e4–22 (+/+, +/−, and −/−) mice were tested in a battery of assays in order to assess the effects of environment on anxiety‐like behavior, motor function, and stereotypy at 8–10 weeks of age. The experimenter was blind to both genotype and rearing conditions until data analysis. The assays were performed in order they are described, but not every cohort was put through every test. See Table 1 for details of each cohort, including the numbers of mice in each experimental group and the tests performed. Both male and female mice were used for all experiments presented in this study.

**Table 1:** Order of behavioral tests for experimental cohorts and number of mice per group

Cohort 1	Cohort 2	Cohort 3	Totals
+/+ Standard: *n* = 11 +/+ Enriched: *n* = 6 +/− Standard: *n* = 8 +/− Enriched: *n* = 7 −/− Standard: *n* = 7 −/− Enriched: *n* = 7	+/+ Standard: *n* = 4 +/+ Enriched: *n* = 14 +/− Standard: *n* = 5 +/− Enriched: *n* = 2 −/− Standard: *n* = 14 −/− Enriched: *n* = 6	+/+ Standard: *n* = 4 +/+ Enriched: *n* = 8 +/− Standard: *n* = 15 +/− Enriched: *n* = 10 −/− Standard: *n* = 4 −/− Enriched: *n* = 3	+/+ Standard: *n* = 19 +/+ Enriched: *n* = 28 +/− Standard: *n* = 28 +/− Enriched: *n* = 19 −/− Standard: *n* = 25 −/− Enriched: *n* = 16
Elevated zero maze	Open field	Rota‐rod	Elevated zero maze: *n* = 13–25 per group
Open field	Elevated zero maze		Open field: *n* = 13–25 per group
Rota‐rod	Hole‐board		Rota‐rod: *n* = 10–25 per group
Grooming			Grooming: *n* = 7–11 per group
Hole‐board			Hole‐board: *n* = 9–21 per group

#### 2.3.1 Zero maze

Mice were introduced into a closed portion of the maze and were given 5 min of free exploration under dim (40–60 lux) illumination. Activity was scored by Ethovision XT 7 (Noldus Information Technologies) using a high‐resolution camera suspended 180 cm above the center of the maze. Tracking profiles were generated by Ethovision XT software and were used to measure the time each mouse spent in the open portions of the maze.

#### 2.3.2 Open field

Activity in the open field was measured over 1 hr in an automated Omnitech Digiscan apparatus (AccuScan Instruments, Columbus, OH). AccuScan software scored the total distance travelled and the time spent in the center of the apparatus.

#### 2.3.3 Rota‐rod

Motor performance was assessed on a steady‐speed rota‐rod (Med‐Associates) set to 20 rotations per minute. Each mouse attempted four trials with an intertrial interval of 30 min. The latency to fall off the apparatus was recorded. If a mouse displayed three successive passive rotations, this was also counted as a fall. Each trial ended after 5 min, and any mouse that successfully remained on the rod at the end of the trial was recorded as a latency of 300 s.

#### 2.3.4 Grooming

Individual animals were acclimated to clean home cages for 5 min prior to filming (MediaRecorder 2; Noldus Information Technologies). Mice were filmed for 10 min, and grooming behavior was hand‐scored using Observer 9 XT (Noldus Information Technologies).

#### 2.3.5 Hole‐board

Mice were allowed 5 min of exploration on a 16‐hole‐board apparatus. Animals were filmed with a digital video camera and hand‐scored for the numbers of nose pokes and the location of each nose poke. Back‐to‐back nose pokes were defined as when the animal made two or more consecutive visits to the same hole.

### 2.4 Statistical analysis

Graphs were produced, and statistical analysis was performed in GraphPad Prism 7. For the rota‐rod data, a repeated‐measures ANOVA was performed and each of the six groups (three genotypes × two rearing conditions) was independently compared with each other group with Tukey’s multiple comparison post hoc test. For all other tests, a two‐way ANOVA for genotype and rearing condition was performed. Significant differences in genotype were followed up with Tukey’s multiple comparison post hoc test. Statistical significance was defined as *p* < 0.05.

## 3 RESULTS

### 3.1 *Shank3* complete knockout mice display repetitive behaviors which are not ameliorated by early environmental enrichment

**Figure 1:**
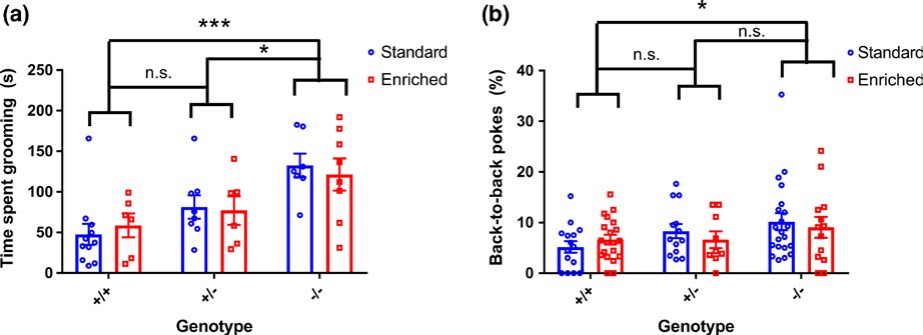
Early environmental enrichment has no effect on restricted and repetitive behaviors in Shank3 ∆e4–22 mice. (a) *Shank3* ∆e4–22^−/−^ mice show elevated rates of grooming compared with both +/+ and *Shank3* ∆e4–22^+/−^ mice (two‐way ANOVA main effect of genotype, *p* < 0.001; Tukey’s multiple comparisons −/− vs. +/+, *p* < 0.001; −/− vs. +/−, *p* < 0.05). There was no effect of rearing condition. *n* = 6–11 per group. (b) *Shank3* ∆e4–22^−/−^ mice perform a higher percentage of back‐to‐back pokes compared with +/+ mice (two‐way ANOVA main effect of genotype, *p* < 0.05, Tukey’s multiple comparisons −/− vs. +/+, *p* < 0.05). *n* = 13–21 per group. *signifies *p* < 0.05, **signifies *p* < 0.01, ***signifies *p* < 0.001, and n.s. stands for not significant

Consistent with our previous reports, the *Shank3* ∆e4–22^−/−^ mice engaged in increased amounts of repetitive self‐grooming, compared to both *Shank3* ∆e4–22^+/−^ and +/+ mice (Figure 1a). There was no effect of environment on the expression of this behavior. Similarly, the *Shank3* ∆e4–22^−/−^ mice engaged in repetitive behavior, in terms of increased back‐to‐back pokes compared to +/+ mice, on the hole‐board task and this phenotype was not affected by the rearing conditions of the mice (Figure 1b).

### 3.2 Early environmental enrichment does not affect spontaneous motor activity, but increases anxiety‐like behavior irrespective of genotype

**Figure 2:**
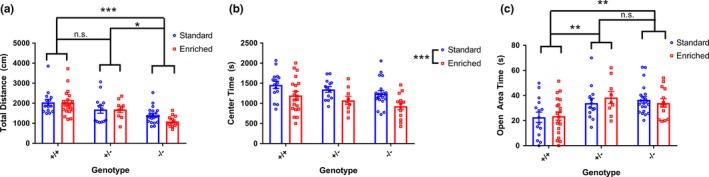
*Shank3* ∆e4–22^−/−^ mice display reduced spontaneous motor activity which is unaffected by rearing condition, but environmental enrichment increases anxiety‐like behavior in all genotypes. (a) *Shank3* ∆e4–22^−/−^ mice are hypoactive in the open field, as total distance travelled is significantly reduced compared with both +/+ and *Shank3* ∆e4–22^+/−^ mice (two‐way ANOVA main effect of genotype, *p* < 0.001; Tukey’s multiple comparisons −/− vs. +/+, *p* < 0.001, −/− vs. +/−, *p* < 0.05). There is no effect of rearing condition on this phenotype. *n* = 9–21 per group. (b) There is no significant effect of genotype on time spent in the center of the open‐field apparatus, but there is a main effect of environment where enriched mice spend significantly less time in the center of the arena (two‐way ANOVA main effect of genotype, *p* < 0.05, but no significant post hoc comparisons; two‐way ANOVA main effect of environment, *p* < 0.001). *n* = 9–21 per group. (c) *Shank3* ∆e4–22^−/−^ and ∆e4–22^+/−^ mice spend more time in the open areas of the elevated zero maze compared with +/+ mice (two‐way ANOVA main effect of genotype, *p* < 0.001; Tukey’s multiple comparisons −/− vs. +/+, *p* = 0.001, +/− vs. +/+, *p* < 0.01). *n* = 13–25 per group. *signifies *p* < 0.05, **signifies *p* < 0.01, ***signifies *p* < 0.001, and n.s. stands for not significant

*Shank3* ∆e4–22^−/−^ mice are hypoactive in the open‐field exploration task, compared to both +/+ and *Shank3* ∆e4–22^+/−^ mice, and there is no effect of rearing condition on this phenotype (Figure 2a). However, contrary to our previous findings, we did not see an effect of genotype on time spent in the center of the open field (Figure 2b). Rather, we observed a main effect of rearing condition, where mice raised in enriched environments spent significantly less time in the center of the arena, regardless of genotype (Figure 2b). We further tested anxiety‐like behavior on the elevated zero maze and found that both *Shank3* ∆e4–22^−/−^ and *Shank3* ∆e4–22^+/−^ mice spend more time in the open areas than +/+ mice, but that this phenotype was not influenced by environmental enrichment (Figure 2c).

### 3.3 Early environmental enrichment has a negative impact on motor performance which is specific to wild‐type mice

**Figure 3:**
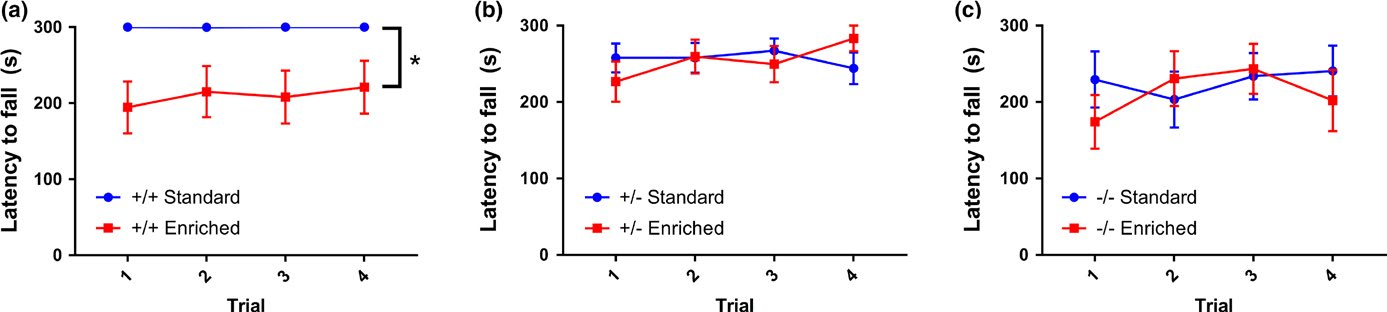
Enriched rearing decreases motor performance in wild‐type mice (a) All +/+ mice raised under standard conditions performed perfectly on the 20 r.p.m. steady‐speed rota‐rod. However, +/+ mice raised in enriched environments performed significantly worse than +/+ raised in standard cages (RMANOVA, main effect of genotype/environment group, *p* < 0.05; Tukey’s multiple comparisons +/+ standard vs. +/+ enriched, *p* < 0.05). *n* = 14–15 per group. (b) There was no statistically significant effect of environment for the +/− mice. *n* = 17–23 per group. (c) There was no statistically significant effect of environment for the −/− mice. *n* = 10–11 per group. *signifies *p* < 0.05, **signifies *p* < 0.01, ***signifies *p* < 0.001, and n.s. stands for not significant

In our previous characterization of the *Shank3* complete knockouts, we found the most severe motor performance deficits on the steady‐speed variation of the rota‐rod. All fifteen of the +/+ mice that were raised in standard conditions performed perfectly throughout all four trials, and this was not true for mice in any other experimental condition (Figure 3a–c). By repeated‐measures ANOVA, there was a significant effect of experimental group on motor performance; however, the only significant differences in post hoc analysis were between +/+ mice raised under standard conditions and +/+ mice raised in enriched environments (Figure 3a).

## 4 DISCUSSION

Our results indicate that raising *Shank3* mutant mice in enriched environments has little effect on their behavior, which is somewhat surprising, given that environmental enrichment has been shown to alleviate behavioral phenotypes in a number of other rodent models of ASDs (Favre et al., 2015; Garbugino et al., 2016; Kerr et al., 2010; Kondo et al., 2008; Lacaria et al., 2012; Lonetti et al., 2010; Nag et al., 2009; Oddi et al., 2015; Restivo et al., 2005; Reynolds et al., 2013; Schneider et al., 2006; Yamaguchi et al., 2017). A summary of previous findings along with findings from the current study is provided in Table 2.

**Table 2:** Summary of key findings from previous studies utilizing environmental enrichment and rodent models of ASD

Reference	Type of ASD Model	Enrichment paradigm	Effect on behavior
Current study	Male and female *Shank3* ∆e4–22^−/−^ mice	Mice were transferred to enriched environments staring at PND 10 and were returned to normal environments on PND 60, when behavioral studies began. Enriched environments consisted of larger (75 × 45 × 25 cm) cages with an assortment of toys that were repositioned daily and completely changed out weekly. Prior to weaning, mice were housed with two dams and two litters per enriched cage	Positive effects: None observed Phenotypes that failed to improve: *Shank3* ∆e4–22^−/−^ mice show elevated rates of self‐grooming that were not affected by housing condition. *Shank3* ∆e4–22^−/−^ mice perform more repetitive nose pokes on the hole‐board test, and this phenotype was not influenced by housing condition. *Shank3* ∆e4–22^−/−^ mice are hypoactive in the open field, and this phenotype was not influenced by housing condition. *Shank3* ∆e4–22^−/−^ and *Shank3* ∆e4–22^+/−^ mice spend more time in the open areas of the elevated zero maze compared with +/+ mice, and this phenotype did not depend on rearing condition. Exacerbations: Enrichment reduced the amount of time mice spent in the center of the open field, regardless of genotype. Enrichment produced deficits on the rota‐rod test specifically in wild‐type mice
Restivo et al. (2005)	Male FMR1‐KO mice on a C57BL/6 background	Starting at PND 21 and lasting for 60 days, enriched mice were housed in groups of three in 35 × 20 × 25 cm cages with an assortment of toys that were changed every 3 days	Positive effects: Thigmotaxis in open field was prevented in enriched FMR1‐KO mice Impaired habituation to objects was prevented in enriched FMR1‐KO mice Phenotypes that failed to improve: Hyperactive locomotion in the open field was not affected by housing condition of the FMR1‐KO mice Exacerbations: Not reported
Kondo et al. (2008)	Hemizygous male and heterozygous female *Mecp2* knockout mice on a mixed C57BL/6 and 129 background	Starting at PND 28 and continuing throughout behavioral testing (at 6–29 weeks), enriched mice were housed in groups of 5–6 and were in larger (size not specified) cages with access to toys that were changed every 2 days	Positive effects: Deficits in rota‐rod performance were prevented in heterozygous females Enrichment improved motor performance on rota‐rod in wild‐type males Phenotypes that failed to improve: Hemizygous males displayed similarly impaired performances on the rota‐rod regardless of housing condition Decreased vertical activity of heterozygous females and hemizygous males was not affected by housing condition Exacerbations: Enriched housing induced a hypolocomotive phenotype in female heterozygous mice
Nag et al. (2009)	*Mecp2* hemizygous hypomorphic male mice (*Mecp2^1lox^*) on a C57BL/6 background	At PND 21, enriched mice were housed in larger (47 × 25 × 21 cm) cages in groups of 4–6. Enriched mice had access to various toys which were exchanged weekly	Positive effects: Enrichment prevented a hypolocomotive phenotype observed in standard‐housed mutant mice Phenotypes that failed to improve: Mutant mice had decreased performance on the accelerating rota‐rod, and this phenotype was not affected by housing condition Enrichment did not significantly improve contextual or cued fear conditioning, which is impaired in mutant mice Exacerbations: Not reported
Kerr et al. (2010)	Hemizygous male *Mecp2* knockout mice on a mixed C57BL/6 and 129 background and hemizygous male *Mecp2* knockout mice on a pure 129 background	PND 21 mice were transferred to enriched environments for two weeks and then were returned to normal conditions. Enriched cages consisted of two connected 30 × 30 cm cages with toys that were changed daily.	Positive effects: Impaired gait in mutants was prevented by enrichment Mutants showed impaired performance on the elevated beam task, and this phenotype was rescued in enriched mutants Mutant mice spent significantly more time in the open areas of the elevated plus maze and enriched housing prevented this phenotype Phenotypes that failed to improve: Mutant mice have impaired survival, and this is not improved by enrichment Exacerbations: Not reported
Lonetti et al. (2010)	Hemizygous male and heterozygous female *Mecp2* knockout mice on a mixed C57BL/6 and 129 background	Mice were transferred to enriched environments staring at PND 10 and were returned to normal environments on PND 60. Hemizygous males were used for tests of motor functions and were testing during the enrichment period (PND 30–60), whereas heterozygous females were used for the other tests, which were performed after PND 60. Enriched environments consisted of larger (44 × 62 × 28 cm) cages with an assortment of toys that were repositioned daily and completely changed out weekly. Prior to weaning, mice were housed with two dams and two litters per enriched cage	Positive effects: Enrichment prolonged survival of hemizygous male mutants, although this was not statistically significant Enrichment prevented impairment on the rota‐rod that was present for mutants raised in standard conditions Heterozygous female mutants raised in standard conditions displayed spatial learning deficits on the Morris Water Maze, but this was prevented in the mice that were reared in enriched environments Female heterozygous mutants raised in standard conditions displayed thigmotaxis in the open field, but mutants raised in enriched conditions did not Phenotypes that failed to improve: Not reported Exacerbations: Not reported
Lacaria et al. (2012)	Male Dp(11)17/+ mice on a C57BL/6 J background	Enriched housing began at PND 21, which consisted of groups of 7–8 mice in larger (27.3 × 22.6 × 48.9 cm) cages with toys that were replaced weekly. Mice were transferred back to standard cages prior to behavior testing	Positive effects: Mutants raised in standard conditions showed impaired (but not statistically significant) social recognition in the partition test, but enriched mutants did not show this phenotype. Mutants raised in standard conditions showed increased aggression in a direct social interaction test and enrichment reduced the amount of contact aggression in these mice. Enrichment increased the amount of nose pokes on the hole‐board test in both wild types and mutants. Mutants in standard housing showed impaired contextual fear conditioning, but this phenotype was prevented by environmental enrichment. Standard‐housed, but not enriched, mutants showed thigmotaxis in the open field. Standard‐housed, but not enriched mutants had decreased entries into the open arms of the elevated plus maze. Enrichment improved motor coordination on the wire‐hang test for both wild types and mutants. Phenotypes that failed to improve: Mutant mice showed increased social dominance in the tube test, and this was not influenced by housing condition. Mutant mice spent less time sniffing social odors, and this was not impacted by housing condition. Mutant mice showed increased repetitive nose pokes on the hole‐board test, and this was not influenced by housing condition. Exacerbations: Enriched housing increased noncontact aggression in the direct social interaction test in both wild types and mutants. Enrichment introduced a hyperactive phenotype in wild‐type mice in the open field
Oddi et al. (2015)	Male FMR1‐KO mice on a FVB background	Enriched mice were housed with an additional nonlactating dam 1 week prior to birth until weaning. After weaning, they were housed under standard conditions	Positive effects: Enrichment reduced the number and duration of PND8 USVs and increased PND8 body weight in both wild types and mutants. Mutants reared in standard conditions were hyperactive in the open field, but enriched mutants were similar to wild type. Mutants reared in standard conditions spent less time interacting with a social stimulus, but enriched mutants were similar to wild type. Mutants reared in standard conditions showed deficits in spontaneous alteration in the T‐maze and in context fear conditioning, but enriched mutants did not Phenotypes that failed to improve: Not reported Exacerbations: Not reported
Garbugino et al. (2016)	Male and female mice lacking the µ‐opioid receptor gene (*Oprm1 −/−*)	Enriched mice were housed with an additional lactating female from approximately 1 week before birth to weaning	Positive effects: Enrichment increased body weight of all mice at PND8, but this normalized by weaning. There was a significant effect of environment such that enriched mice (of both genotypes) spent more time interacting with the social stimulus in the juvenile social approach‐avoidance test compared to standard‐housed mice. For male mice specifically, there was also an effect of enrichment on adult social behavior. While mutant mice spent less time investigating an intruder mouse regardless, enrichment increased the investigation time in both wild types and mutants. Phenotypes that failed to improve: While enrichment decreased the number of PND8 USVs in wild‐type mice, mutants had decreased numbers of USVs compared with wild type, and this was not affected by enrichment. Exacerbations: Not reported
Reynolds et al. (2013)	Male BTBR inbred mouse strain	The mice were placed in enriched housing in groups of 8 at 7 weeks of age for 30 days. The enrichment cage was a three‐floor dog kennel with various toys that were changed every 5 days	Positive effects: BTBR mice self‐groom significantly more than C57BL/6 mice, and this phenotype was rescued by environmental enrichment Phenotypes that failed to improve: Not reported Exacerbations: Not reported
Yamaguchi et al. (2017)	Male mice exposed prenatally to VPA	One week after weaning, mice were in enriched environments for four weeks. This consisted of a larger cage (65 × 35 × 30 cm) filled with toys that were repositioned twice per week	Positive effects: VPA‐exposed mice housed in standard conditions spent more time in the closed arms of the elevated plus maze compared with controls, but enriched VPA‐exposed mice were similar to controls. VPA‐exposed mice housed in standard conditions spent less time sniffing a stimulus in a social interaction test compared to controls, but enriched VPA‐exposed mice were similar to controls. VPA‐exposed mice housed in standard conditions showed deficits in novel object recognition, but enriched VPA‐exposed mice were similar to controls. Phenotypes that failed to improve: VPA‐exposed mice were hypoactive, reared less, and had fewer center crossings in the open field irrespective of environmental condition. Exacerbations: Not reported
Schneider et al. (2006)	Male rats prenatally exposed to valproic acid (VPA)	Enriched rats underwent multisensory stimulation from PND 7–21 and further enriched housing from PND 22–35. Multisensory stimulation involved exposing pups to various temperatures and textures for approximately 25 min per day. Enriched housing consisted of 12 rats housed in a large aquarium (60 × 60 × 40 cm) filled with toys that were changed every 2 days	Positive effects: Increased thermal nociceptive threshold and reduced mechanical allodynia were prevented in enriched VPA rats Diminished acoustic prepulse inhibition was prevented in enriched VPA rats Hyperactivity and increased repetitive movements in open field were prevented in enriched VPA rats Reduced exploratory activity (rearing, hole‐poking) was prevented in enriched VPA rats Enriched rearing increased pinning behavior during social play and social exploration in both VPA and control rats Enriched rearing increased time spent in the open arms of the elevated plus maze in both VPA and control rats Phenotypes that failed to improve: Not reported Exacerbations: Not reported
Favre et al. (2015)	Male rats prenatally exposed to valproic acid (VPA)	Rats were enriched from PND 23–123. Enriched rats were in larger cages in groups of six and were given access to various toys. Enriched environments were either “predictable” or “unpredictable,” which simply meant whether or not the toys were changed at all. In the “unpredictable” condition the toys were changed twice weekly	Positive effects: VPA‐exposed rats housed in standard conditions had increased fear conditioning responses, but VPA‐exposed rats raised in “unpredictable” enriched environments were similar to controls. However, VPA‐exposed rats raised in “predictable” enriched environments had an impairment in fear conditioning. Phenotypes that failed to improve: VPA‐exposed rats had increased repeated entries in the Y‐maze task regardless of housing condition. Exacerbations: VPA‐exposed rats housed in the “predictable” enriched environments had higher sociability in the three‐chamber test compared to control rats housed in similar conditions. However, this appears to be mostly because of a decrease in sociability in the enriched controls compared to standard‐housed controls. VPA‐exposed rats housed in “predictable” enriched environments spent more time in the open arms of the elevated plus maze compared to the other groups

Particularly, striking was that although we modeled our enrichment paradigm after the one described in a previous study that found positive effects of enrichment on a mouse model of Rett syndrome (Lonetti et al., 2010), including increased time spent in the center of the open field and increased motor performance on the rota‐rod, we found that enrichment negatively impacted both of these measurements in our mouse model. This might suggest that a “one‐size fits all” approach may not work for behavioral intervention with patients with different genetic mutations, but the translational value of the findings in these studies is not immediately clear. One possible explanation for the conflicting findings in the two studies is the different clinical presentations of Rett syndrome and Phelan–McDermid syndrome in humans; while patients with *SHANK3* mutations typically display signs of neurological impairments at birth (Phelan & McDermid, 2012), patients with *MECP2* mutations typically present after 12–16 months (Neul et al., 2010). Another possible explanation is that because in our attempt to remain blind to experimental condition, we removed mice from the enriched environments prior to behavior testing and this may have had an anxiogenic effect. The previous study utilizing the mouse model of Rett syndrome kept the mice in enriched environments throughout testing. A potential follow‐up experiment could specifically examine the effect of transferring mice back to standard cages following the enrichment paradigm.

However, there are many other possible explanations for the negative results presented in the current study, including varying methods of enrichment and differences in genetic backgrounds used in previous studies, as well as different methods of assessing behavioral phenotypes. While the negative results presented do not exclude the possibility that a different form of enrichment would be beneficial to our mouse model, it would be unreasonably time consuming to try all the different variations of enrichment. Regardless, the lack of effect of environment suggests that Shank3 mutations are highly penetrant and underscores the need for molecularly targeted pharmaceutical intervention. Still, environmental enrichment remains promising for other forms of ASD and many other brain‐related disorders (reviewed in Nithianantharajah & Hannan, 2006).

We did not observe any sex differences in this study or in our previous characterization of the Shank3 knockout mice. Although human males are more likely to be diagnosed with ASD compared to females, there is no apparent sex bias for *SHANK3* mutations (De Rubeis et al., 2018; Sarasua et al., 2014). Another consistent finding from both this study and our previous characterization of the Shank3 ∆e4–22 mice is a lack of a strong phenotype in heterozygous animals. Heterozygous animals performed statistically the same as wild‐type animals on all assays in this study, except for the increased time spent in the open areas of the elevated zero maze, but humans with SHANK3‐related ASD are missing just one copy of the gene. Heterozygous mice displayed an intermediate phenotype on many of the behavioral assays, but again, performed statistically the same as wild types. It remains to be determined whether humans are more sensitive to smaller changes in dosage of the SHANK3 protein, whether the highly controlled environment of the laboratory prevents the expression of ASD‐like phenotypes in heterozygotes, or whether the current methods used for assessing behavior in rodents prevent us from detecting more subtle changes most relevant to ASD.
